# miR-93-5p enhances migration and invasion by targeting RGMB in squamous cell carcinoma of the head and neck

**DOI:** 10.7150/jca.43854

**Published:** 2020-04-06

**Authors:** Shuiting Zhang, Yanjuan He, Chao Liu, Guo Li, Shanhong Lu, Qiancheng Jing, Xiyu Chen, Huiling Ma, Diekuo Zhang, Yunyun Wang, Donghai Huang, Pingqing Tan, Jie Chen, Xin Zhang, Yong Liu, Yuanzheng Qiu

**Affiliations:** 1 Department of Otolaryngology Head and Neck Surgery, Xiangya Hospital, Central South University, 87 Xiangya Road, Changsha, Hunan 410008, People's Republic of China.; 2 Otolaryngology Major Disease Research Key Laboratory of Hunan Province, 87 Xiangya Road, Changsha, Hunan 410008, People's Republic of China.; 3 Department of Hematology, Xiangya Hospital, Central South University, 87 Xiangya Road, Changsha, Hunan 410008, People's Republic of China.; 4 Department of Otolaryngology Head and Neck Surgery, Changsha Central Hospital,161 Shaoshan Road, University of South China, Changsha, Hunan 410004, People's Republic of China.; 5 Department of Head and Neck Surgery, Hunan Cancer Hospital, The Affiliated Tumor Hospital of Xiangya Medical School, Central South University, 283 Tongzipo Road, Changsha, Hunan 410013, People's Republic of China.; 6 Clinical Research Center for Pharyngolaryngeal Diseases and Voice Disorders in Hunan Province, 87 Xiangya Road, Changsha, Hunan 410008, People's Republic of China.

**Keywords:** miR-93-5p, squamous cell carcinoma of the head and neck, RGMB, epithelial-mesenchymal transition

## Abstract

Invasion and metastasis represent the primary causes of therapeutic failure in patients diagnosed with squamous cell carcinoma of the head and neck (SCCHN). Therefore, disease prediction and inhibition of invasion and metastasis are critical for enhancing the survival of patients with SCCHN. Our previous study revealed that increased expression of miR-93-5p is associated with poor prognosis in SCCHN; however, the mechanism underlying the oncogenic functions of miR-93-5p in SCCHN migration and invasion remains unclear. Using qPCR analyses, transwell assays, and scratch tests, we demonstrated that expression of ectopic miR-93-5p induced the migration and invasion of SCCHN, and this was accompanied by corresponding alterations in biomarkers and transcription factors specific for epithelial-mesenchymal transition (EMT). Luciferase reporter assays were used to demonstrate that miR-93-5p directly targeted the 3' UTR of RGMB, and we further found that the tumor-promoting functions of miR-93-5p were partly mediated by targeting RGMB, whose downregulation also promoted the migration and invasion of SCCHN. Overall, our results indicate that miR-93-5p acts as an oncogene in the regulation of migration and invasion by suppressing RGMB in SCCHN. These findings provide novel evidence that miR-93-5p may serve as a valuable predictive biomarker and potential intervention target in patients with SCCHN.

## Introduction

Squamous cell carcinoma of the head and neck (SCCHN) is the sixth most common tumor worldwide, with approximately 650,000 cases being reported annually over the past few decades 1, 2. Despite advances in therapeutic strategies, the 5-year survival rate for SCCHN has not significantly improved, and this lack of improvement is due to late diagnosis, frequent loco-regional recurrences at the primary site, and cervical lymph node metastasis 3, 4. New targeted agents against invasion and metastasis could provide crucial countermeasures to improve the prognosis of patients with SCCHN. Thus, specific molecular signatures and an improved understanding of the underlying mechanisms of SCCHN migration and invasion are urgently required to improve therapeutic efficacy and to aid in the design of more effective treatment strategies against SCCHN.

The discovery of miRNAs has provided a new avenue for understanding the regulatory mechanisms underlying gene expression. miRNAs constitute a family of small non-coding RNAs that are approximately 22 nucleotides in length 5, and these molecules can play oncogenic or tumor suppressor roles during tumorigenesis by influencing malignant biological behaviors such as proliferation, apoptosis, radioresistance, chemoresistance, and metastasis^6^-^9^. miR-93-5p, an miRNA within the miR-106b-25 cluster, has been reported to be dysregulated in various types of cancer such as cervical, breast, and bladder cancers, as well as nasopharyngeal carcinoma 10-13. Our previous study demonstrated that miR-93-5p is upregulated in SCCHN tissues, and this upregulation is closely related to the clinicopathological parameters of T status, lymph node metastasis, and clinical stage 14. Additionally, it has been previously reported that high expression of miR-93-5p is associated with poor prognosis 14. Despite these findings, the underlying mechanisms regulating the functional involvement of miR-93-5p in SCCHN migration and invasion remain unclear.

In this study, through the use of transwell assays and scratch tests, we determined that miR-93-5p clearly enhanced the migration and invasion of SCCHN. Mechanistically, miR-93-5p promoted the process of epithelial-to-mesenchymal transition (EMT) in SCCHN cells. Additionally, we found that the tumor suppressor, RGMB, was directly targeted by miR-93-5p and was partially involved in the migration and invasion mediated by miR-93-5p. Overall, our results indicated that miR-93-5p exerts critical regulatory control of migration and invasion by suppressing RGMB. These findings provide valuable clues that will allow researchers to further elucidate the molecular mechanisms underlying migration and invasion in SCCHN.

## Materials and Methods

### Cell culture

SCCHN Tu686 and CAL27 cell lines were kindly gifted by Dr. Zhuo G. Chen (Emory University School of Medicine, Atlanta, USA) and Dr. Joseph Califano (University of California, San Diego, USA), respectively. The human tongue squamous carcinoma cell line Tcal8113 was obtained from the Cell Bank of Type Culture Collection of the Chinese Academy of Sciences. The human hypopharyngeal carcinoma cell line FaDu was purchased from ATCC. SCCHN 6-10B and 5-8F cells were obtained from the Cell Center of Central South University, Changsha, China. Tu686 cells were maintained in DMEM/F12 medium, Fadu and CAL27 cells were grown in DMEM basic medium, and 6-10B, 5-8F, and Tcal8113 cells were cultured in RPMI 1640 medium. All medium types were supplemented with 10% FBS, 100 μg/mL streptomycin, and 100 units/mL penicillin (Gibco, Grand Island, NY), and the cells were cultured at 37 °C under 5% CO_2_. Cells within the logarithmic growth phase were used for subsequent experiments.

### TCGA data analysis

Expression profiles for 522 SCCHN and 44 noncancerous samples were obtained from The Cancer Genome Atlas (TCGA) database.

### RNA isolation and quantitative reverse-transcriptase PCR (qRT-PCR) analysis

Total RNA was isolated from cell lines using the Trizol reagent (Invitrogen, CA, USA), and first strand cDNA was generated using the All-in-One^TM^ miRNA or mRNA cDNA synthesis kit (GeneCopoeia Inc., MD, USA) in a 25 μL reaction system containing 1 μg of total RNA. A 0.5 μL aliquot of cDNA was amplified using All-in-One^TM^ miRNA or mRNA Mix (GeneCopoeia Inc., MD, USA) in each 20 μL reaction system. The amplification procedure was performed using the ABI 7300 Fast Real-Time PCR system (Applied Biosystems), and the reaction parameters included an initial step at 95°C for 10 min, 40 cycles of 95°C for 10 seconds, 62.5°C for 20 seconds, and 72°C for 15 seconds. Expression values were calculated using the 2^-ΔΔCT^ method and normalized to an internal control (U6 for miRNA and GAPDH for mRNA). The primers used for the qRT-PCR are listed in Supplementary Table S1.

### Transfection

SCCHN cells were transfected with the miR-93-5p mimics, the miR-93-5p inhibitors, or with negative control (NC) miRNA (Genepharma, Suzhou, China) using si-Mate^TM^ (Genepharma) according to the manufacturer's protocol. Additionally, to establish a cell line that stably expressed miR-93-5p, Tu686 cells were infected with lentivirus expressing miR-93-5p (GeneCopoeia Inc., MD, USA), and the infected cells were selected in the medium containing 3 μg/mL puromycin.

### Wounding-healing and transwell invasion assay

The specific wound-healing and invasion assays were performed as previously described 15-17. For the wound healing assay, a sterile micropipette tip was used to scratch SCCHN cells that were at an 80-90% subconfluence, and the cells were then cultured in serum-free medium for another 48 h. Cells were photographed under a microscope, and the results were assessed as percent of scratch closure. For the invasion assay, the transfected cells (1~2 × 10^4^) were seeded in the top chamber in 100 µL of serum-free medium (Corning, NY, USA) with Matrigel (BD Biosciences, San Diego, CA, USA), and the insert was placed in a 24-well culture plate. Complete medium (500 µL) containing 10% FBS was added to the lower chamber. After 48 h, the invaded cells located on the lower side of the insert were fixed and stained. Five random fields per well were observed, and the cells were counted under the microscope.

### Colony formation assay

A total of 200 cells were seeded into 6-well plates and incubated for 14 days. Next, the cells were fixed with methanol for 15 min and subsequently stained with 0.1% crystal violet for 10 min. The colonies were assessed using Image J v1.8.0 (positive colony was defined as > 50 cells).

### Western blotting

SCCHN cells were lysed for protein extraction using RIPA lysis buffer. After protein quantification, equal amounts of proteins were loaded and separated in 8-12% SDS-PAGE gels and then transferred onto PVDF membranes (Millipore, Bedford, MA, USA). Next, the membrane was sealed with 5% skimmed milk and incubated with corresponding primary antibodies as previously described 18. GAPDH was used as a loading control. Target protein bands were immuno-detected with enhanced chemiluminescent substrate. The antibodies used in this study are listed in Supplementary Table S2.

### Immunofluorescence assays

Cells were seeded into a 12-well plate containing sterile slides and then cultured for 24 h. The slides were then fixed in 4% paraformaldehyde for 15 min, permeabilized using 0.2% Triton X-100 for 10 min, blocked with 5% BSA for 1 h, and then incubated overnight with a rabbit primary antibody specific for E-cadherin (Santa Cruz, CA) or a mouse antibody specific for Vimentin (Proteintech Group, Wuhan, China). Subsequently, Alexa Fluor 488 goat anti-rabbit IgG (H + L) and Alexa Fluor 594 goat anti-mouse IgG (Jackson Immuno Research, WestGrove, PA) were used for secondary detection under dark conditions, and the cells were counterstained with DAPI (Sigma, St. Louis, MO, USA). Images were observed using a Leica fluorescence microscope (Wetzlar, Germany).

### Xenograft tumor model

Cell aliquots (200 μL) were injected into 4-week-old male immune-deficient BALB/c nude mice (n = 5 each group) that were purchased from Hunan SJA Laboratory Animal Co., Ltd. (Changsha, Hunan, China). Tumor sizes were monitored, and the volumes were calculated as 0.5 × length × width^2^. After approximately four weeks, the mice were euthanized by cervical dislocation, and tumor samples were then harvested. All animal research protocols were approved by the Animal Ethics Committee of Central South University (Changsha, Hunan, China).

### Luciferase reporter assays

Luciferase assays were performed 48 h after co-transfection with 50 nM of either miR-93-5p mimics or NC oligos in combination with 200 ng of wild-type or mutated 3'-UTR RGMB plasmids (GeneCopoeia Inc., MD, USA) using the Dual-Luciferase assay kit (Promega) according to the manufacturer's protocol. Firefly and Renilla luciferase activities were assessed using the Dual-Luciferase® Reporter Assay System (Promega).

### Statistical analysis

Descriptive statistics and means ± SD were obtained using the SPSS 17.0 statistical software program. Two-sample t tests and ANOVA were performed to analyze the significance of differences between groups. P-values of less than 0.05 were regarded as statistically significant.

## Results

### Overexpression of miR-93-5p enhances SCCHN migration and invasion

**Figure 1:**
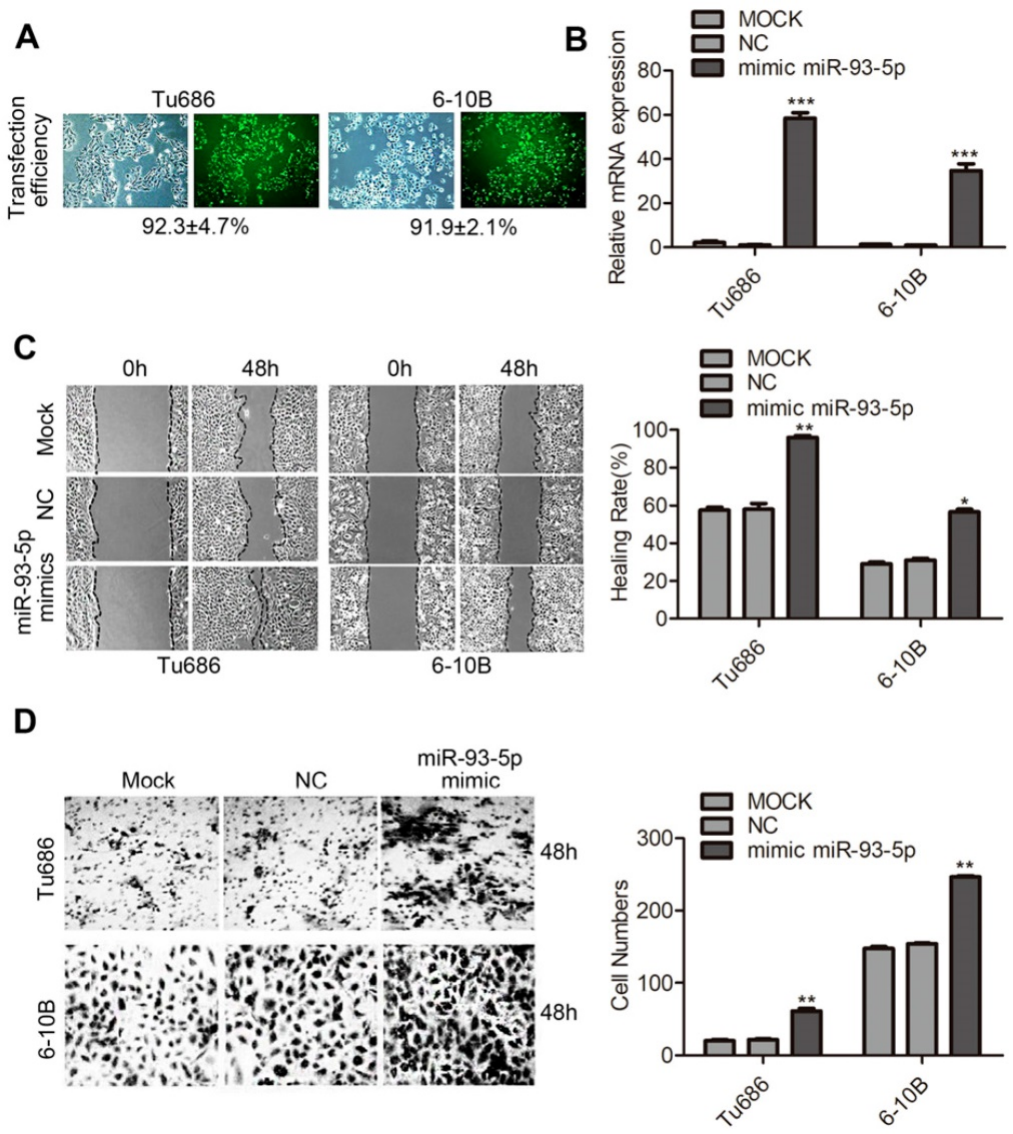
** Overexpression of miR-93-5p enhances SCCHN migration and invasion. A,** Representative fluorescence images were obtained under a fluorescence microscope. **B,** The expression of miR-93-5p was detected using qRT-PCR after transfection with a miR-93-5p-mimic. **C,** The migration ability of cells was examined using a scratch test, and the healing rate was calculated. **D,** Representative images from inserts were obtained for transwell invasion assays, and the invaded cells were quantified. Data are presented as the mean ± SD. Student's unpaired *t*-test*,* *, *P* <0.05; **, *P* < 0.01; ***, *P* < 0.001.

To assess the tumor-promoting function of miR-93-5p in the context of SCCHN, we first investigated its expression in 6 SCCHN cell lines. Results based on qRT-PCR analyses indicated that the expression of miR-93-5p was significantly higher in Tcal8113 and lower in 6-10B (Supplementary Fig. S1), and based on this, we selected these cell lines for loss and gain of function experiments, respectively. The cell line Tu686 that exhibited modest miR-93-5p expression was also included in the functional experiments. Next, a transfection efficiency of >90% was observed for the miR-93-5p-mimic through the use of fluorescence microscopy (Fig. 1A), and the expression of miR-93-5p was significantly upregulated by 40-60-fold after transfection with the miR-93-5p-mimic in SCCHN Tu686 and 6-10B cells (Fig. 1B). Migration and invasion capacities were then investigated using transwell and wound-healing assays in SCCHN cells. As shown in Fig. 1C, compared to mock and negative control cells, cell migration and invasion were significantly increased in miR-93-5p overexpressing cells. Additionally, plate cloning analyses revealed that miR-93-5p did not affect the proliferative capacity of SCCHN cells (Supplementary Fig. S2A and S2B). We also used lentiviral vectors to engineer Tu686 cells to stably overexpress miR-93-5p, facilitating the establishment of xenograft tumors in athymia mice. Our results indicated that cell proliferation was unaffected *in vivo* following the upregulation of miR-93-5p (Supplementary Fig. S2C-F). Collectively, these data extended and reinforced our studies by demonstrating that miR-93-5p enhances the migratory and invasive abilities of SCCHN cells.

### Knockdown of miR-93-5p suppresses SCCHN migration and invasion

**Figure 2:**
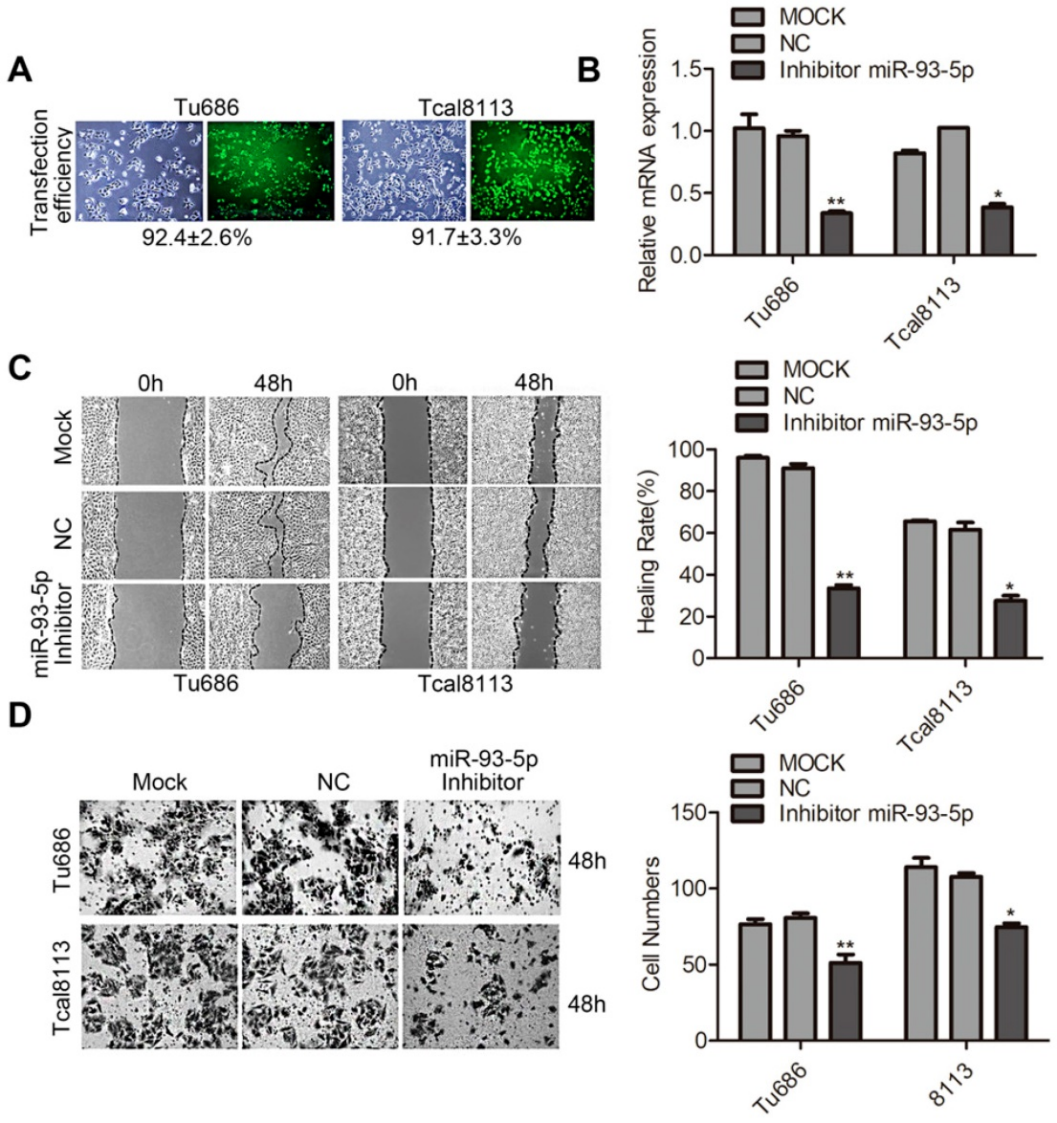
** Knockdown of miR-93-5p suppresses SCCHN migration and invasion. A** and** B,** The transfection efficiency of miR-93-5p-inhibitor (**A**) and expression of miR-93-5p (**B**) were determined using a fluorescence microscope and qRT-PCR analyses, respectively. **C,** Scratch tests using Tu686 and Tcal8113 cells revealed that cell migration ability was inhibited by the miR-93-5p-inhibitor. **D,** Representative images of cell invasion were obtained from transwell assays, and the invaded cells were imaged 48 h post incubation to allow for quantification of cell numbers. All data are represented as the mean ± SD. Statistical analysis was performed using Student's *t*- test. *, *P* <0.05; **, *P* < 0.01.

To further explore the function of miR-93-5p in SCCHN, loss of function experiments were performed using Tu686 and Tcal8113 cell lines. A transfection efficiency of > 90% was obtained using the miR-93-5p-inhibitor (Fig. 2A), and miR-93-5p expression was downregulated by more than 70% in both Tu686 and Tcal8113 cells (Fig. 2B). Additionally, the wound closure rates and invaded cell numbers for miR-93-5p-suppressed cells were attenuated compared to those of the control, suggesting that miR-93-5p inhibition suppressed the migratory and invasive ability of SCCHN cells (Fig. 2C and 2D).

### Upregulation of miR-93-5p promotes EMT in SCCHN

**Figure 3:**
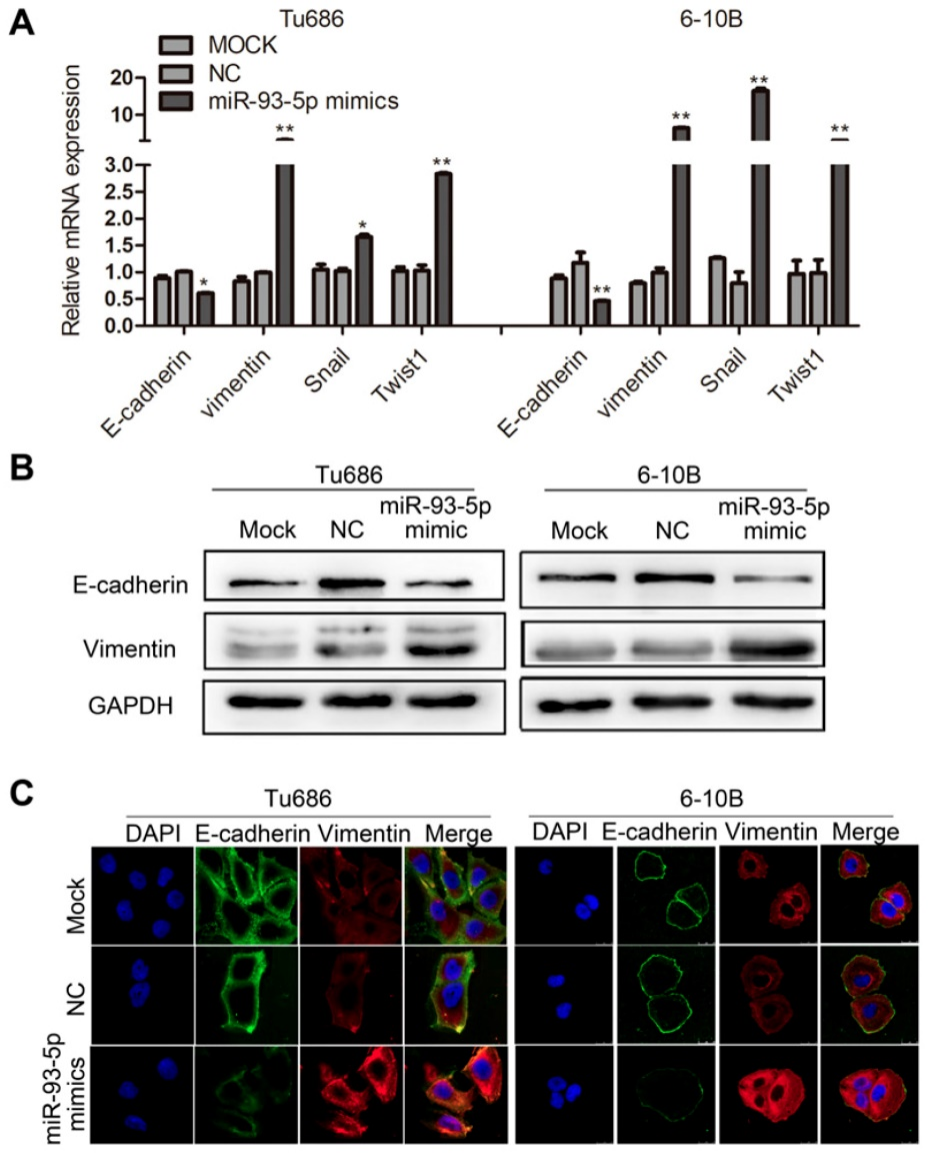
** Upregulation of miR-93-5p promotes EMT in SCCHN. A,** qPCR analysis was used to quantify the expression of the EMT markers of E-cadherin and Vimentin and the related transcription factors snail and Twist1 in SCCHN cells after transfection with miR-93-5p-mimic. GAPDH was used as a loading control. **B** and** C**, Representative immunoblotting analyses (**B**) and immunofluorescence staining (**C**) were conducted to show the expression level of E-cadherin and Vimentin in Tu686 and 6-10B cells. All data are presented as mean ± SD. Student's unpaired *t*-test. *, *P* < 0.05; **, *P* < 0.01.

EMT is a key developmental program underlying the malignant biological behaviors of invasion and metastasis, and this process can be characterized by specific biomarkers 19. We first analyzed the correlation between miR-93-5p expression and the mRNAs of EMT markers in SCCHN cells. Our results indicated that miR-93-5p overexpression in Tu686 and 6-10B cells resulted in decreased mRNA expression of the epithelial marker E-cadherin and increased mRNA expression of the mesenchymal marker Vimentin, and these changes were accompanied by the promotion of the EMT transcription factors Snail and Twist1 (Fig. 3A). These results were confirmed by western blot analysis (Fig. 3B). Additionally, immunofluorescent staining of these cells revealed that the expression of E-cadherin was inhibited and the expression of Vimentin was upregulated following overexpression of miR-93-5p in Tu686 and 6-10B cells (Fig. 3C). These data indicated that upregulation of miR-93-5p promoted EMT in SCCHN.

### miR-93-5p inhibition impedes EMT in SCCHN

**Figure 4:**
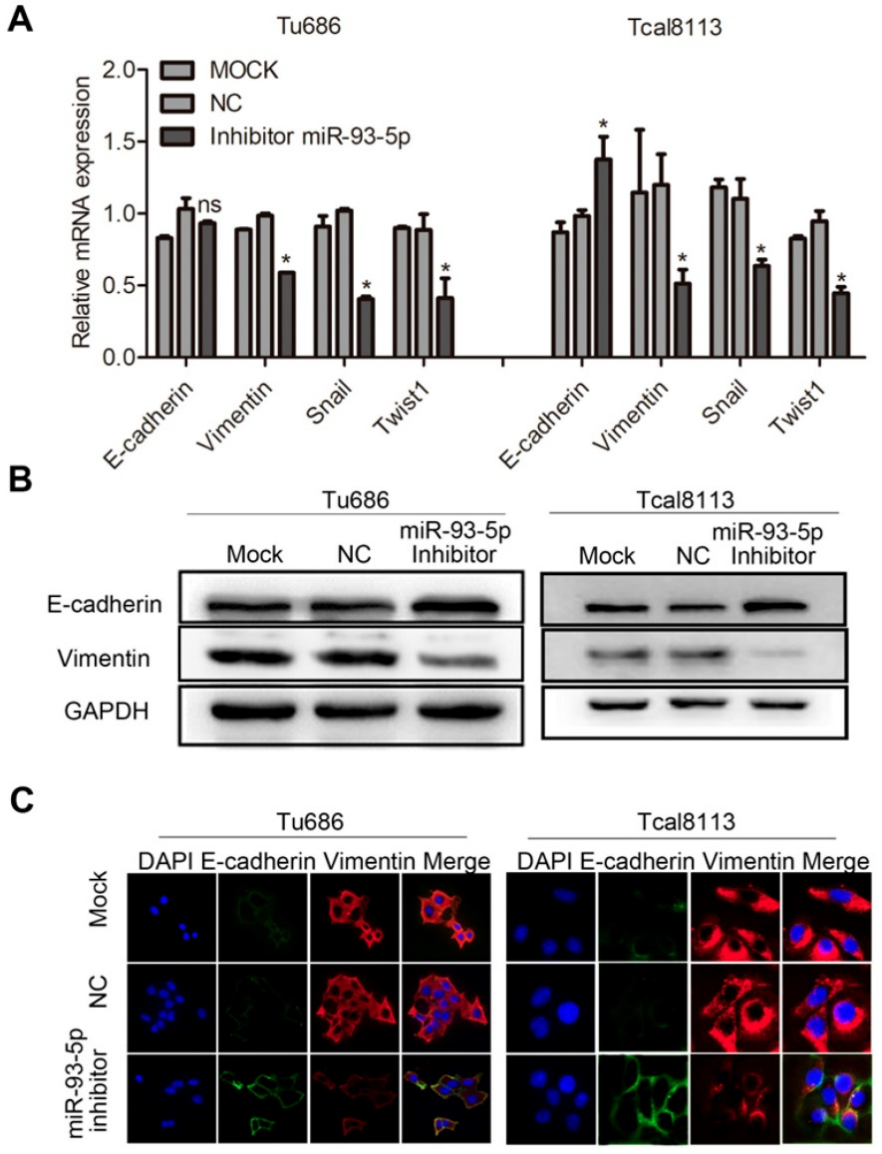
** miR-93-5p inhibition impedes EMT in SCCHN. A,** The mRNA levels of E-cadherin, Vimentin, and EMT-related transcription factors in cells after miR-93-5p inhibition were quantified by qRT-PCR. **B,** The protein levels of E-cadherin and Vimentin were analyzed by western blotting using the indicated antibodies. **C,** Immunofluorescence assay of EMT markers is shown. Data are presented as the mean ± SD. *P*-values were calculated using Student's *t*-test. *, *P* < 0.05; **, *P* < 0.01; ns, not significant.

We also investigated the effect of miR-93-5p inhibition in the context of EMT in Tu686 and Tcal8113 cell lines. Following transfection with a miR-93-5p-inhibitor, qRT-PCR analyses revealed that downregulation of miR-93-5p significantly increased the mRNA expression of E-cadherin in Tcal8113 cell lines and decreased the mRNA expression of Vimentin in both Tu686 and Tcal8113 cell lines, and these changes were accompanied by the inhibition of the EMT transcription factors, Snail and Twist1 (Fig. 4A). Additionally, we used western blotting and immunofluorescent analysis to confirm that miR-93-5p suppression elevated the levels of E-cadherin and suppressed the levels of Vimentin in both Tu686 and Tcal8113 cells (Fig. 4B and 4C).

### RGMB is a direct target of miR-93-5p

**Figure 5:**
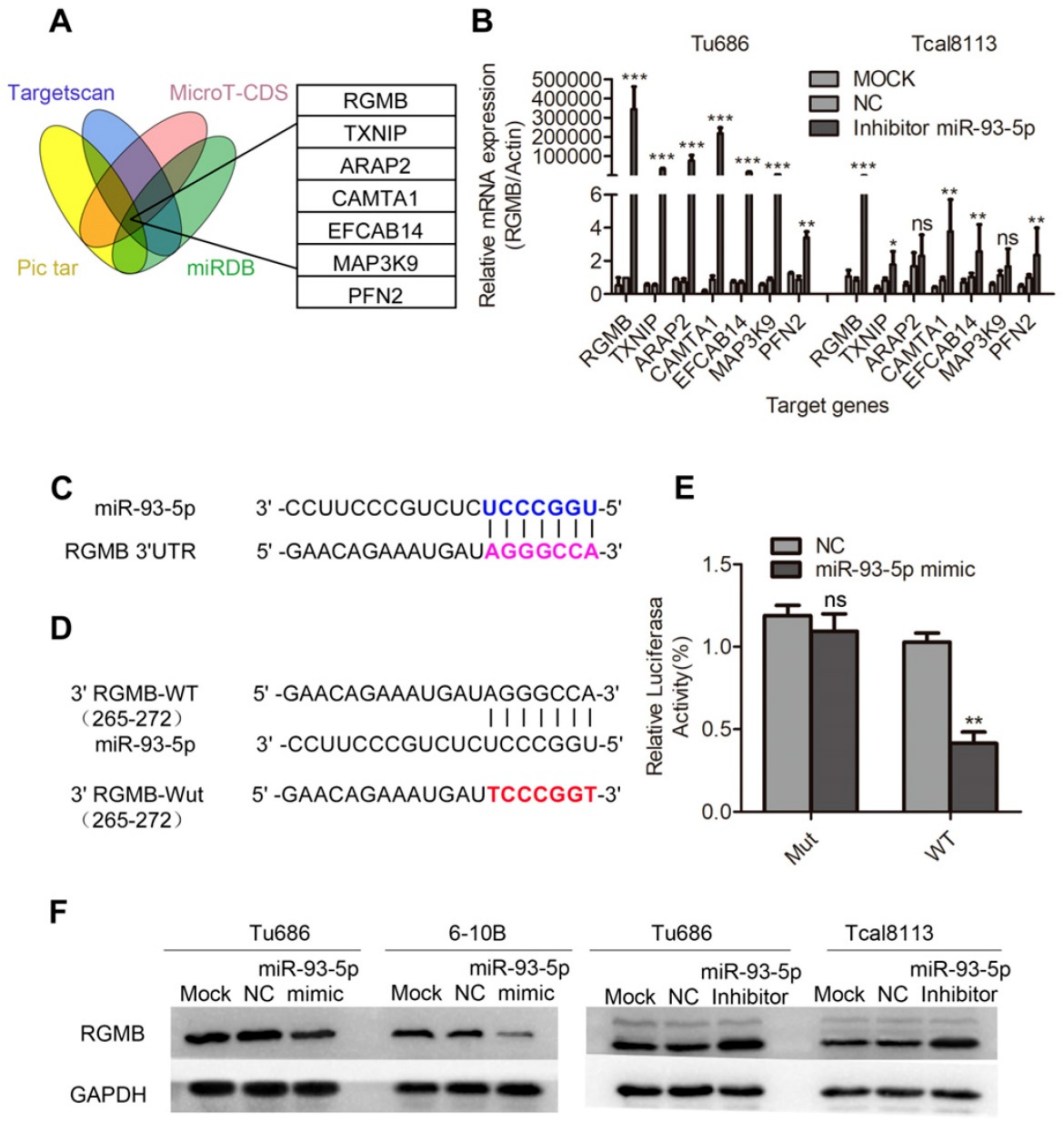
** RGMB is a direct target of miR-93-5p. A,** Seven candidate genes were obtained through the use of four algorithms (TargetScan, Pictar, miRBD, and MicroT-CDS). **B,** Results of qRT-PCR analyses of the mRNA expression of potential target genes, including RGMB, after miR-93-5p inhibition in Tu686 and Tcal8113 cells. **C,** Predicted binding of miR-93-5p to the 3'-UTR of RGMB.** D** and** E,** Dual luciferase reporter assay revealed the interaction of miR-93-5p and its targeting sequence in the RGMB 3'-UTR. Tu686 cells were transfected with the wild-type (WT) or mutated (Mut) target site of the RGMB 3'-UTR (**D**) and miR-93-5p-mimic or NC for 48 h. The luciferase activity was determined and is presented as relative activity to the corresponding NC (**E**). **F,** RGMB protein expression was measured by western blotting in SCCHN cells that were transfected with the miR-93-5p mimic or inhibitor. Data are presented as the mean ± SD. Student's unpaired *t*-test. **, *P* < 0.01; ***, *P* < 0.001; ns, not significant.

To further explore the target genes of miR-93-5p, we applied four algorithms (miRNAPicTar/www.pictar.org, TargetScan/www.targetscan.org, MicroT-CDS/diana.imis.athe na-innovation.gr, and miRNABD/sysbio.suda.edu.cn/MiRNA-BD) to predict the mRNA targets. Seven promising candidate genes, including RGMB, were obtained (Fig. 5A). Based on our findings, we inhibited the expression of miR-93-5p in the Tu686 and Tcal8113 cell lines, and RGMB was selected based on the observation that its expression ranked at the top of the gene list (Fig. 5B). Sequence alignment revealed that the RGMB gene possessed a potential miR-93-5p binding sequence in its 3'UTR region (Fig. 5C). We further confirmed the direct binding of miR-93-5p to the 3'UTR of RGMB mRNA using the luciferase reporter assay (Fig. 5D and 5E) and validated the dysregulation of RGMB protein by miR-93-5p using a panel of SCCHN cell lines (Fig. 5F). Additionally, we investigated the relationship between the expression of miR-93-5p and RGMB based on TCGA SCCHN data. Our results revealed that the expression level of miR-93-5p was upregulated in tumor samples, and the expression of RGMB was upregulated in normal samples (Supplementary Fig. S3A, B). Additionally, an inverse relationship between the expression of miR-93-5p and that of RGMB was observed in TCGA SCCHN samples (Supplementary Fig. S3C). Taken together, these results indicated that RGMB is a direct target of miR-93-5p.

### RGMB is partially involved in the migration and invasion of SCCHN caused by miR-93-5p

**Figure 6:**
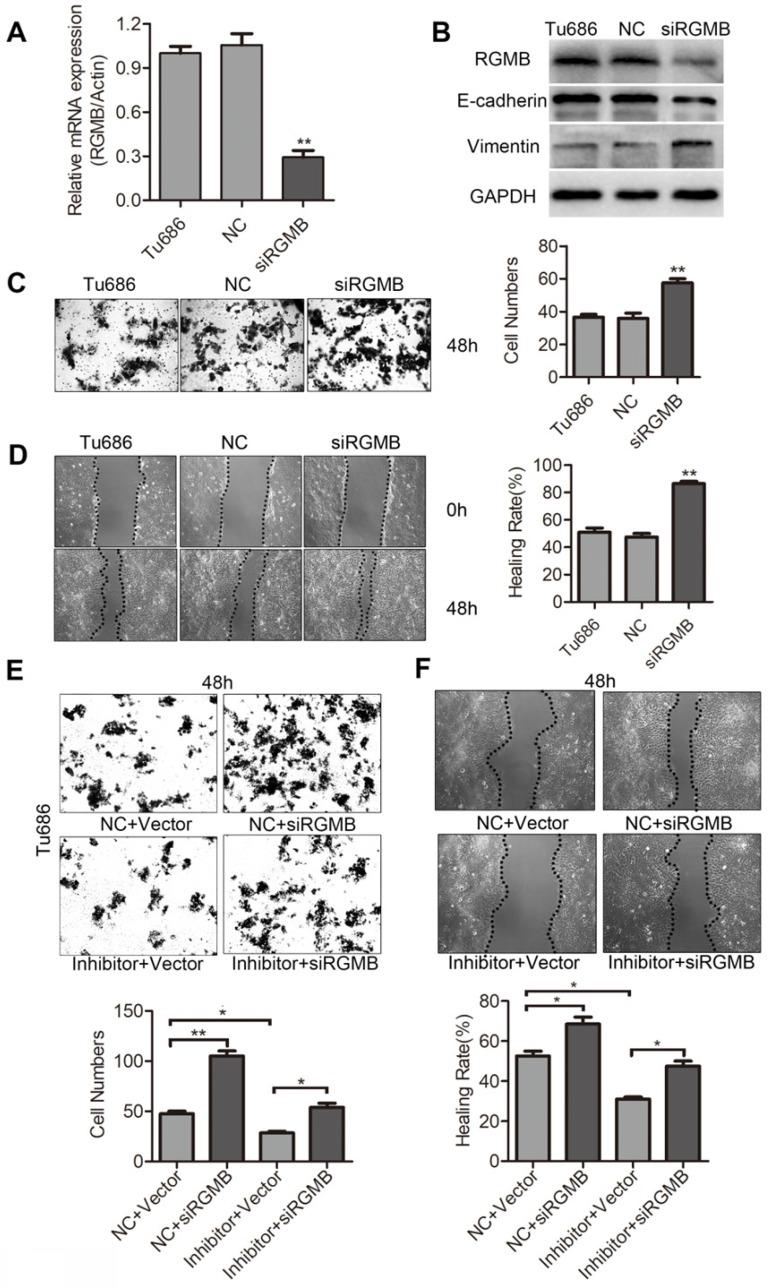
** RGMB is partially involved in the invasion of SCCHN that is mediated by miR-93-5p. A,** RGMB mRNA expression was analyzed using qRT-PCR in Tu686 cells after siRGMB treatment. **B,** Western blotting showed the expression of E-cadherin and Vimentin in Tu686 cells and revealed that RGMB was suppressed in cells successfully. **C** and** D,** Following knockdown of RGMB in Tu686 cells, transwell invasion (**C**) and wound-healing (**D**) assays were performed. **E** and** F,** Cells transfected with miR-93-5p mimics or NC were subsequently treated with siRGMB or negative control. Wound closure (**E**) and transwell staining (**F**) were evaluated for 48 h post culture in Tu686 cells. Data are presented as the mean ± SD. *P*-values were calculated using the Student's *t*-test. *, *P* <0.05; **, *P* < 0.01.

To finally ascertain if downregulation of RGMB is responsible for miR-93-5p-mediated migration and invasion of SCCHN, an RGMB siRNA was constructed and subsequently expressed in Tu686 cells. Initially, we examined the role of RGMB expression in the migration and invasion of Tu686 cells. Our results indicated that the expression of RGMB mRNA and protein was markedly decreased following transfection with siRGMB (Fig. 6A and 6B). Additionally, RGMB suppression significantly increased the protein levels of Vimentin and reduced those of E-cadherin in Tu686 cells (Fig. 6B). Further results indicated that downregulation of RGMB significantly increased the migration and invasion in Tu686 cells (Fig. 6C and 6D). Tu686 cells were next transfected with RGMB siRNA to counteract the miR-93-5p-inhibitor-induced enhanced expression of RGMB. As expected, RGMB restored the inhibitory effect on cell migration and invasion caused by the miR-93-5p-inhibitor in Tu686 cells (Fig. 6E and 6F). These findings demonstrated that RGMB is involved in miR-93-5p-mediated migration and invasion.

## Discussion

Accumulating evidence indicates that the aberrant expression of miRNA plays a crucial role in cancer development 20. Based on the findings from our previous studies, in our current study, we characterized the effects of miR-93-5p on the migration and invasion of SCCHN cells. Additionally, our rescue experiments in combination with the direct targeting of miR-93-5p to the predicted 3'UTR sequences of RGMB further substantiated the presence of a miR-93-5p/RGMB regulatory axis in SCCHN. Our study highlights the functional role of miR-93-5p-modulated post-transcriptional repression of RGMB that could provide a new mechanism for the regulation of SCCHN migration and invasion.

Invasion and metastasis are important hallmarks of malignancy and are also a major cause of therapeutic failure. A number of researchers have demonstrated that miRNAs are involved in the invasion and metastasis of various cancers, including SCCHN. For example, miR-98 can inhibit invasion and metastasis of SCCHN by downregulating MTDH, and miR-654-5p can target GRAP to promote metastasis of SCCHN through the Ras/MAPK signaling pathway 21, 22. miR-93-5p has been mapped to intron 13 of the MCM7 gene, and this could allow it to function in cooperation with the oncogenic miR-106b-25 cluster to regulate numerous malignant biological behaviors. miR-93-5p has been demonstrated to directly target DAB2 to promote cell migration and invasion in prostate cancer 23, and it can also regulate cell migration and invasion by suppressing PTEN through the PI3K/Akt pathway in breast cancer 24. In SCCHN, salivary miR-93-5p may provide a potential biomarker for the post-radiation monitoring of SCCHN, and miR-93-5p has been shown to possess oncogenic function in laryngeal squamous cell carcinoma by targeting cyclin G2 25, 26. Our previous study also revealed that increased expression of miR-93-5p is associated with poor prognosis in SCCHN 14. In our current study, we used scratch healing and transwell invasion assays to confirm that miR-93-5p could promote SCCHN cell migration and invasion *in vitro* and that this simultaneously activated the EMT program.

EMT is a process that involves the trans-differentiation of epithelial cells into motile mesenchymal cells, and this process acts as a key developmental program in the malignant biological behaviors of invasion and metastasis 27. During tumorigenesis, cells lose their initial characteristics and are transformed into mesenchymal fibroblast-like cells. This morphological transition of EMT fosters the ability of cells to break away from their originating tissue and allows them to migrate and invade into the surrounding environment 28, 29. Additionally, tumor epithelial cells that undergo EMT share many characteristics with stem cells, and this significantly complicates systemic therapies used to treat related metastatic diseases 29, 30. Investigating the mechanisms underlying EMT may therefore provide an effective approach to combat SCCHN. miRNAs have been previously reported to be associated with cancer EMT processes 31. Several prior studies have demonstrated that miR-93-5p can induce EMT in breast, lung, and liver cancers, as well as nasopharyngeal carcinoma 32-35;however, a few studies paradoxically found that miR-93-5p inhibits EMT in breast cancer by targeting MKL-1, STAT3, and stem cell regulatory genes 36, 37. These findings highlight the complicated relationship between miR-93-5p and EMT within different types of cancers. In this study, we revealed that miR-93-5p could promote EMT in SCCHN, and this was accompanied by corresponding alterations in the EMT markers E-cadherin, Vimentin, Snail, and Twist1.

miRNAs typically exert their functions by downregulating the expression of target genes, and it is well-established that a given miRNA can interact with multiple targets. In our study, we combined four algorithm-based analyses to investigate promising candidate targets of miR-93-5p, and we found that RGMB was a major target gene of this miRNA. RGMB, also known as Dragon, was originally identified from a genomic screen for genes that are regulated by the transcription factor DRG11 in the embryonic dorsal root ganglion 38, 39. The biological functions of RGMB have only recently begun to emerge. Li *et al*. reported that knockdown of RGMB provides a favorable environment for proliferation and adhesion in breast cancer cells through the BMP signaling pathway 40, and Ying *et al*. found that elevated expression of RGMB promotes tumorigenesis in colorectal cancer 41. Despite these previous findings, studies examining the role of RGMB in cancer metastasis and EMT remain scarce, particularly those related to SCCHN. To the best of our knowledge, the present study is the first to demonstrate that RGMB knockdown inhibits SCCHN migration and invasion *in vitro*, indicating that RGMB serves as a tumor suppressor in SCCHN metastasis. We also found that downregulation of RGMB abrogates increased migration and invasion induced by miR-93-5p inhibition, suggesting that RGMB is a major target of miR-93-5p. Additionally, although miR-93-5p induced EMT changes in SCCHN, we repeated the online prediction to confirm that EMT genes such as E-cadherin and Vimentin etc were not direct targets of miR-93-5p. Additionally, restored expression of RGMB partially reversed the expression of EMT genes, which also indicated that these EMT genes were not directly targeted by miR-93-5p.

In summary, in the current study, we revealed the oncogenic functions for miR-93-5p in SCCHN migration and invasion *in vitro*. Additionally, we identified RGMB as a direct target of miR-93-5p and demonstrated that RGMB is essential for mediating the tumor-promoting effects of miR-93-5p in SCCHN cells. Based on our findings, this study demonstrates that targeting the miR-93-5p/RGMB axis may provide a potential novel strategy for SCCHN treatment.

## Supplementary Material

Supplementary figures and tables.

## References

[1] Jing Q, Li G, Chen X, Liu C, Lu S, Zheng H (2019). Wnt3a promotes radioresistance via autophagy in squamous cell carcinoma of the head and neck. Journal of cellular and molecular medicine.

[2] Read ML, Modasia B, Fletcher A, Thompson RJ, Brookes K, Rae PC (2018). PTTG and PBF Functionally Interact with p53 and Predict Overall Survival in Head and Neck Cancer. Cancer Res.

[3] Wu X, Yeerna H, Goto Y, Ando T, Wu VH, Zhang X (2019). Metformin Inhibits Progression of Head and Neck Squamous Cell Carcinoma by Acting Directly on Carcinoma-Initiating Cells. Cancer Res.

[4] Huang D, Qiu Y, Li G, Liu C, She L, Zhang D (2018). KDM5B overexpression predicts a poor prognosis in patients with squamous cell carcinoma of the head and neck. Journal of Cancer.

[5] El Bezawy R, Cominetti D, Fenderico N, Zuco V, Beretta GL, Dugo M (2017). miR-875-5p counteracts epithelial-to-mesenchymal transition and enhances radiation response in prostate cancer through repression of the EGFR-ZEB1 axis. Cancer Lett.

[6] Srivastava AK, Banerjee A, Cui T, Han C, Cai S, Liu L (2019). Inhibition of miR-328-3p Impairs Cancer Stem Cell Function and Prevents Metastasis in Ovarian Cancer. Cancer Res.

[7] van Kampen JGM, van Hooij O, Jansen CF, Smit FP, van Noort PI, Schultz I (2017). miRNA-520f Reverses Epithelial-to-Mesenchymal Transition by Targeting ADAM9 and TGFBR2. Cancer Res.

[8] Li G, Liu Y, Su Z, Ren S, Zhu G, Tian Y (2013). MicroRNA-324-3p regulates nasopharyngeal carcinoma radioresistance by directly targeting WNT2B. Eur J Cancer.

[9] Croset M, Pantano F, Kan CWS, Bonnelye E, Descotes F, Alix-Panabieres C (2018). miRNA-30 Family Members Inhibit Breast Cancer Invasion, Osteomimicry, and Bone Destruction by Directly Targeting Multiple Bone Metastasis-Associated Genes. Cancer Res.

[10] Sun XY, Han XM, Zhao XL, Cheng XM, Zhang Y (2019). MiR-93-5p promotes cervical cancer progression by targeting THBS2/MMPS signal pathway. European review for medical and pharmacological sciences.

[11] Li JP, Xiang Y, Fan LJ, Yao A, Li H, Liao XH (2019). Long noncoding RNA H19 competitively binds miR-93-5p to regulate STAT3 expression in breast cancer. Journal of cellular biochemistry.

[12] Juracek J, Peltanova B, Dolezel J, Fedorko M, Pacik D, Radova L (2018). Genome-wide identification of urinary cell-free microRNAs for non-invasive detection of bladder cancer. Journal of cellular and molecular medicine.

[13] Tang JF, Yu ZH, Liu T, Lin ZY, Wang YH, Yang LW (2014). Five miRNAs as novel diagnostic biomarker candidates for primary nasopharyngeal carcinoma. Asian Pacific journal of cancer prevention: APJCP.

[14] Li G, Ren S, Su Z, Liu C, Deng T, Huang D (2015). Increased expression of miR-93 is associated with poor prognosis in head and neck squamous cell carcinoma. Tumour biology: the journal of the International Society for Oncodevelopmental Biology and Medicine.

[15] Qin Y, Wang J, Zhu G, Li G, Tan H, Chen C (2019). CCL18 promotes the metastasis of squamous cell carcinoma of the head and neck through MTDH-NF-kappaB signalling pathway. Journal of cellular and molecular medicine.

[16] Liu C, Li G, Ren S, Su Z, Wang Y, Tian Y (2017). miR-185-3p regulates the invasion and metastasis of nasopharyngeal carcinoma by targeting WNT2B in vitro. Oncology letters.

[17] Zhao XG, Hu JY, Tang J, Yi W, Zhang MY, Deng R (2019). miR-665 expression predicts poor survival and promotes tumor metastasis by targeting NR4A3 in breast cancer. Cell death & disease.

[18] She L, Qin Y, Wang J, Liu C, Zhu G, Li G (2018). Tumor-associated macrophages derived CCL18 promotes metastasis in squamous cell carcinoma of the head and neck. Cancer cell international.

[19] Dang TT, Esparza MA, Maine EA, Westcott JM, Pearson GW (2015). DeltaNp63alpha Promotes Breast Cancer Cell Motility through the Selective Activation of Components of the Epithelial-to-Mesenchymal Transition Program. Cancer Res.

[20] Rupaimoole R, Slack FJ (2017). MicroRNA therapeutics: towards a new era for the management of cancer and other diseases. Nat Rev Drug Discov.

[21] Tan H, Zhu G, She L, Wei M, Wang Y, Pi L (2017). MiR-98 inhibits malignant progression via targeting MTDH in squamous cell carcinoma of the head and neck. American journal of cancer research.

[22] Lu M, Wang C, Chen W, Mao C, Wang J (2018). miR-654-5p Targets GRAP to Promote Proliferation, Metastasis, and Chemoresistance of Oral Squamous Cell Carcinoma Through Ras/MAPK Signaling. DNA and cell biology.

[23] Yang K, Li YW, Gao ZY, Xiao W, Li TQ, Song W (2019). MiR-93 functions as a tumor promoter in prostate cancer by targeting disabled homolog 2 (DAB2) and an antitumor polysaccharide from green tea (Camellia sinensis) on their expression. International journal of biological macromolecules.

[24] Li N, Miao Y, Shan Y, Liu B, Li Y, Zhao L (2017). MiR-106b and miR-93 regulate cell progression by suppression of PTEN via PI3K/Akt pathway in breast cancer. Cell death & disease.

[25] Greither T, Vorwerk F, Kappler M, Bache M, Taubert H, Kuhnt T (2017). Salivary miR-93 and miR-200a as post-radiotherapy biomarkers in head and neck squamous cell carcinoma. Oncology reports.

[26] Xiao X, Zhou L, Cao P, Gong H, Zhang Y (2015). MicroRNA-93 regulates cyclin G2 expression and plays an oncogenic role in laryngeal squamous cell carcinoma. International journal of oncology.

[27] Singh M, Yelle N, Venugopal C, Singh SK (2018). EMT: Mechanisms and therapeutic implications. Pharmacol Ther.

[28] Bastid J (2012). EMT in carcinoma progression and dissemination: facts, unanswered questions, and clinical considerations. Cancer Metastasis Rev.

[29] Fukuda K, Takeuchi S, Arai S, Katayama R, Nanjo S, Tanimoto A (2019). Epithelial-to-Mesenchymal Transition Is a Mechanism of ALK Inhibitor Resistance in Lung Cancer Independent of ALK Mutation Status. Cancer Res.

[30] Mani SA, Guo W, Liao MJ, Eaton EN, Ayyanan A, Zhou AY (2008). The epithelial-mesenchymal transition generates cells with properties of stem cells. Cell.

[31] Musavi Shenas MH, Eghbal-Fard S, Mehrisofiani V, Abd Yazdani N, Rahbar Farzam O, Marofi F (2019). MicroRNAs and signaling networks involved in epithelial-mesenchymal transition. Journal of cellular physiology.

[32] Smith AL, Iwanaga R, Drasin DJ, Micalizzi DS, Vartuli RL, Tan AC (2012). The miR-106b-25 cluster targets Smad7, activates TGF-beta signaling, and induces EMT and tumor initiating cell characteristics downstream of Six1 in human breast cancer. Oncogene.

[33] Qu MH, Han C, Srivastava AK, Cui T, Zou N, Gao ZQ (2016). miR-93 promotes TGF-beta-induced epithelial-to-mesenchymal transition through downregulation of NEDD4L in lung cancer cells. Tumour biology: the journal of the International Society for Oncodevelopmental Biology and Medicine.

[34] Ji C, Liu H, Yin Q, Li H, Gao H (2017). miR-93 enhances hepatocellular carcinoma invasion and metastasis by EMT via targeting PDCD4. Biotechnology letters.

[35] Lyu X, Fang W, Cai L, Zheng H, Ye Y, Zhang L (2014). TGFbetaR2 is a major target of miR-93 in nasopharyngeal carcinoma aggressiveness. Molecular cancer.

[36] Xiang Y, Liao XH, Yu CX, Yao A, Qin H, Li JP (2017). MiR-93-5p inhibits the EMT of breast cancer cells via targeting MKL-1 and STAT3. Experimental cell research.

[37] Liu S, Patel SH, Ginestier C, Ibarra I, Martin-Trevino R, Bai S (2012). MicroRNA93 regulates proliferation and differentiation of normal and malignant breast stem cells. PLoS genetics.

[38] Samad TA, Srinivasan A, Karchewski LA, Jeong SJ, Campagna JA, Ji RR (2004). DRAGON: a member of the repulsive guidance molecule-related family of neuronal- and muscle-expressed membrane proteins is regulated by DRG11 and has neuronal adhesive properties. The Journal of neuroscience: the official journal of the Society for Neuroscience.

[39] Liu W, Chen B, Wang Y, Meng C, Huang H, Huang XR (2018). RGMb protects against acute kidney injury by inhibiting tubular cell necroptosis via an MLKL-dependent mechanism. Proc Natl Acad Sci U S A.

[40] Li J, Ye L, Sanders AJ, Jiang WG (2012). Repulsive guidance molecule B (RGMB) plays negative roles in breast cancer by coordinating BMP signaling. Journal of cellular biochemistry.

[41] Shi Y, Chen GB, Huang XX, Xiao CX, Wang HH, Li YS (2015). Dragon (repulsive guidance molecule b, RGMb) is a novel gene that promotes colorectal cancer growth. Oncotarget.

