# Supplementary material for: miR-93-5p enhances migration and invasion by targeting RGMB in squamous cell carcinoma of the head and neck
Source: J Cancer. 2020 Apr 6;11(13):3871–81. doi: 10.7150/jca.43854 (PMC7171485; doi:10.7150/jca.43854)
Supplement: Supplementary file 1 — Supplementary figures and tables. [file jcav11p3871s1.pdf]

Table S1 The sequence of primers

Gene	Primer sequence
E-cadherin	
Forward	5'-TCC ATT TCT TGG TCT ACG CC -3
Reverse	5'-CAC CTT CAG CCA ACC TGT TT-3
Vimentin	
Forward	5'-TGG CAC GTC TTG ACC TTG AA-3'
Reverse	5'-GGT CAT CGT GAT GCT GAG AA -3'
Twist1	
Forward	5'-AGC AAG ATT CAG ACC CTC AA-3'
Reverse	5'-AGC AAG ATT CAG ACC CTC AA-3'
Snail	
Forward	5'-TTT ACC TTC CAG CAG CCC TA-3'
Reverse	5'-GGA CAG AGT CCC AGA TGA GC-3'
RGMB	
Forward	5'-TCC CCT AGA TGG CGG AGA AA-3
Reverse	5'-AAC GGC AGA GTT CAG GTG AG -3
TXNIP	
Forward	5'-CCG CCT CCT GCT TGA AAC TA-3
Reverse	5'-GTC TCT TGA GTT GGC TGG CT-3
ARAP2	
Forward	5'-CAA GGC TGG GAG AGA CGC-3
Reverse	5'-TTG CTG CAC AGT CCT TCA CA-3
CAMTA1	
Forward	5'-TCC CGG AGA GTA GTG AGA CC-3
Reverse	5'-TTC GCC CAC TCC TTC TTG TC-3
EFCAB14	
Forward	5'-TCA TTC CGT CAC CTT CAG CC-3
Reverse	5'-CAA GCT CTC TGG TTC TGG CA-3
MAP3K9	
Forward	5'-CAA GCC CCA CCA TCA TTC CT-3
Reverse	5'-TTC TCA CGT CTC TGA GGG GA-3
PFN2	
Forward	5'-AGC TGC TAG GCA GAC TGT TA-3
Reverse	5'-TTA AGT GTG CCT CCG TGG AC-3
GAPDH	
Forward	5'-TCC AAA ATC AAG TGG GGC GA-3'
Reverse	5'-AGT AGA GGC AGG GAT GAT GT-3'

Table S2 The antibody information

E-cadherin	Cat.3195T<br>1:1000 ~1:2000 dilution, Cell Signaling Technology, Danvers, MA
Vimentin	Cat.10366-1-AP<br>1:500 ~1:2000 dilution, Proteintech Group, Wuhan, China
RGMB	Cat.ab191916<br>1:1000 ~1:2000 dilution, Abcam, Cambridge, MA
GAPDH	Cat.10494-1-AP<br>1:500 ~1:2000 dilution, Proteintech Group, Wuhan, China
Rabbit IgG	Cat.14708S<br>1:2000 dilution, Cell Signaling Technology, Danvers, MA
Mouse IgG	Cat.14709S<br>1:2000 dilution, Cell Signaling Technology, Danvers, MA
Alexa Fluor 594<br>Mouse IgG	Cat.115-585-003<br>1:1000 ~1:2000 dilution, Jackson Immuno Research, West Grove, PA
Alexa Fluor<br>488 Rabbit IgG	Cat.111-545-003<br>1:1000 ~1:2000 dilution, Jackson Immuno Research, West Grove, PA

A

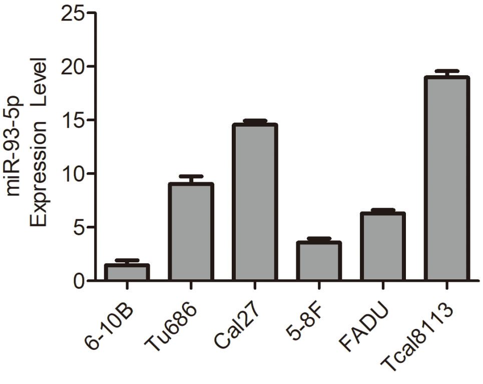

miR-93-5p Expression Level
25
20
15
10
5
0
6-10B
Tu686
Cal27
5-8F
FADU
Tcal8113

Fig. S1

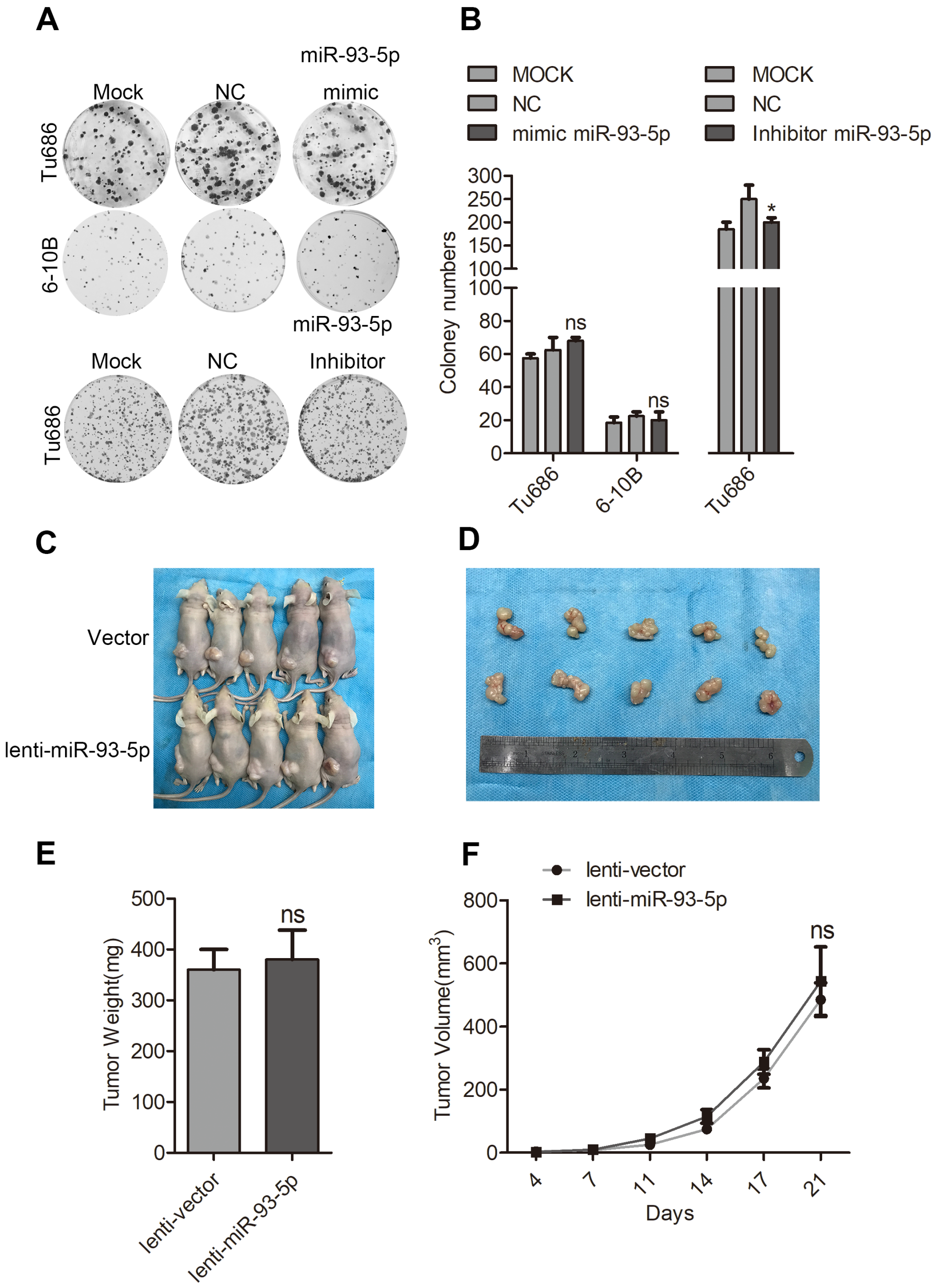
A
Mock
NC
miR-93-5p mimic
Tu686
6-10B
miR-93-5p Inhibitor
B
MOCK
NC
mimic miR-93-5p
Inhibitor miR-93-5p
Coloney numbers
ns
*
Tu686
6-10B
C
Vector
lenti-miR-93-5p
D
E
Tumor Weight(mg)
lenti-vector
lenti-miR-93-5p
F
Tumor Volume(mm3)
Days

Fig. S2

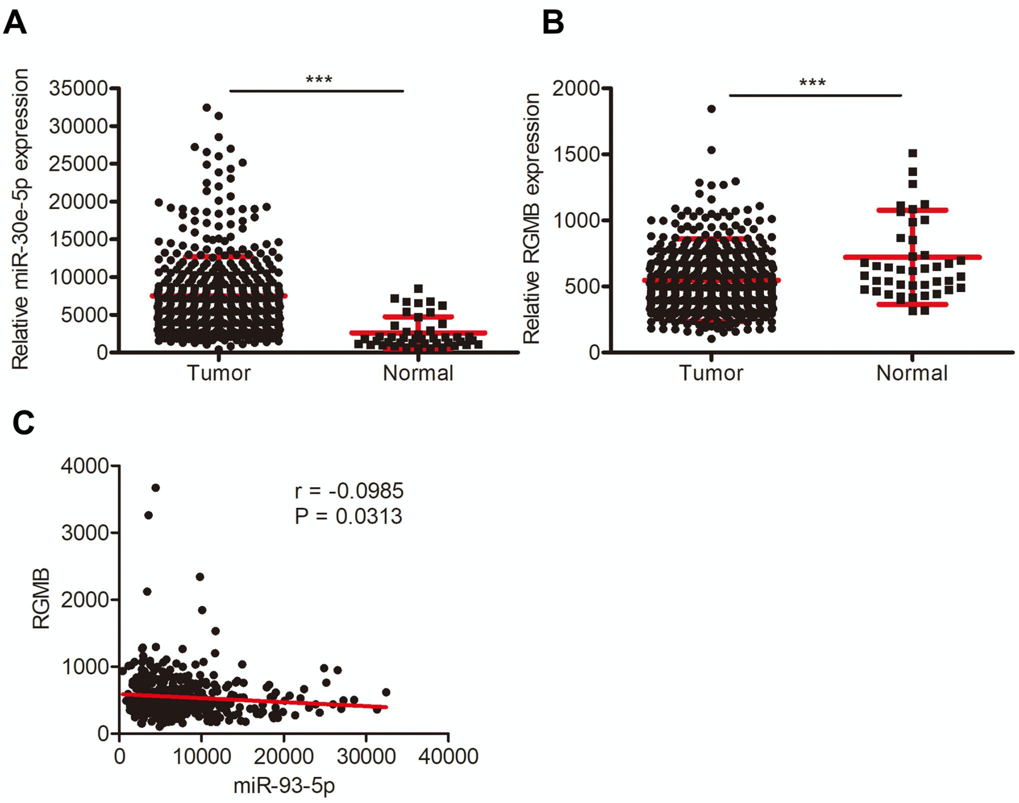
A
B
C
Relative miR-30e-5p expression
35000
30000
25000
20000
15000
10000
5000
0
***
Tumor
Normal
Relative RGMB expression
2000
1500
1000
500
0
***
Tumor
Normal
RGMB
4000
3000
2000
1000
0
r = -0.0985
P = 0.0313
0
10000
20000
30000
40000
miR-93-5p

Fig. S3.
